# Antegrade mini-percutaneous flexible ureteroscopy versus retrograde ureteroscopy for treating impacted proximal ureteric stones of 1–2 cm: A prospective randomised study

**DOI:** 10.1080/2090598X.2020.1769385

**Published:** 2020-08-23

**Authors:** Omar Elgebaly, Hussein Abdeldayem, Faisal Idris, Alaa Elrifai, Ahmed Fahmy

**Affiliations:** Department of Urology, Faculty of Medicine, Alexandria University, Alexandria, Egypt

**Keywords:** Ureteroscopy, laser, mini percutaneous, impacted, proximal ureteric stone

## Abstract

**Objectives:**

To prospectively assess the safety and effectiveness of antegrade mini-percutaneous (miniperc) ureteroscopy (URS) and compare it with the conventional retrograde URS (RURS) approach in treating impacted proximal ureteric stones of 1–2 cm.

**Patients and methods:**

The study included 60 patients admitted to the Department of Urology, Alexandria Main University Hospital, presenting with impacted proximal ureteric stones of 1–2 cm. Patients were randomly divided into two groups: Group A, were treated with RURS using a semi-rigid or flexible ureteroscope to access the stone; and Group B, were treated by antegrade miniperc URS, were a 14-F renal tract was obtained to pass a ureteric access sheath, then a flexible ureteroscope was used going downwards to the stone. Holmium laser was used for stone fragmentation. A JJ stent was inserted in all cases. Follow-up with non-contrast computed tomography was performed after 2 weeks.

**Results:**

Both groups were comparable in terms of patient demographics and stone criteria. The stone-free rate was significantly higher in Group B (83.3%) compared to Group A (60%). The mean (SD) operative time was significantly shorter in Group A vs Group B, at 64.7 (±17.7) vs 112.0 (±15.3) min; while the mean lithotripsy time was comparable between the groups. The mean radiation exposure time was significantly less in Group A (11 s) compared to Group B (200 s). Both groups where comparable concerning minor complications, with no major complications.

**Conclusion:**

Antegrade miniperc flexible URS is safe and more effective than RURS for treating large impacted proximal ureteric stones.

**Abbreviations:**

ESWL: extracorporeal shockwave lithotripsy; KUB: plain abdominal radiograph of the kidneys, ureters and bladder; miniperc: mini-percutaneous; PCNL: percutaneous nephrolithotomy; PCS: pelvi-calyceal system; SFR: stone-free rate; (R)URS: (retrograde) ureteroscopy

## Introduction

A large impacted upper ureteric stone describes stones of >1 cm that lie above the lower border of the forth lumber vertebra and below the PUJ. There is no established definition for the term ‘impacted stone’, but it is generally accepted that it refers to a stone that causes hydronephrosis, as it remains stationery causing obstruction for >6 weeks. Due to oedema surrounding the stone, it prevents passage of dye below it during a contrast study and prevents the passage of a guidewire during ureteroscopy (URS) [1–3]. Different modalities have been reported for managing this category of stones including: extracorporeal shockwave lithotripsy (ESWL), retrograde URS (RURS), antegrade approach, laparoscopy, and rarely open surgery [4]. RURS is now considered the first-line procedure for treating upper third ureteric stones, with an overall stone-free rate (SFR) of 81% (range 77–85%) for stones of >1 cm [4]. A common problem with this approach is retrograde stone retropulsion during fragmentation, with an incidence of 28–60%, hindering the SFR and increasing the need for auxiliary procedures [5,6]. Moreover, stone impaction with surrounding mucosal oedema narrows the field of vision, and thus increases the risk of complications such as perforation and instrument damage [7]. The antegrade approach is another treatment option for large impacted upper ureteric stones, which can avoid the drawbacks of the retrograde approach. However, it has its own limitations as regard the invasive nature and tract formation. We used a mini-percutaneous (miniperc) tract to minimise this limitation. In the present study, we aimed to prospectively compare the safety and effectiveness of treating large impacted upper ureteric stones between the two approaches, i.e., antegrade miniperc URS and conventional RURS.

## Patients and methods

The study was performed prospectively and included 60 patients admitted to the Department of Urology, Alexandria Main University Hospital, from February 2018 to May 2019. All the patients presented with solitary large impacted proximal ureteric stones of 1–2 cm. They were randomly divided into two equal groups (30 patients in each). Group A, treated with RURS and holmium laser stone fragmentation; and Group B, treated by antegrade miniperc URS using the same method of stone fragmentation. Preoperatively, all patients underwent evaluation by history, physical examination, laboratory investigations (full blood and urine tests), and radiological investigations including plain abdominal radiograph of the kidneys, ureters and bladder (KUB) and non-contrast CT to assess stone criteria. Informed medical consent was signed by all patients. We used the closed envelope method for randomisation.

Inclusion criteria:

- Patients with upper third ureteric stones (located below the PUJ and above the lower border of L4).
- Adult age group, aged ≥18 years.
- Stones >1 cm in maximum dimension.

While patients with one or more of the following criteria were excluded from the study:

- Untreated UTI.
- Pregnant females.
- Presence of distal obstruction to the stone.
- Presence of associated renal stones.
- Stone size >2 or <1 cm in maximum dimension.

### Group A, RURS

Under general anaesthesia, all patients were positioned in the classic dorsal lithotomy position in a head down position. The URS was performed using either a semi-rigid URS (9.5-F sheath, 8-F tip, 6-F working channel; Karl Storz SE & Co. KG, Tuttlingen, Germany) or flexible URS (Flex X2, 7.5 F; Karl Storz). The ureteroscope was inserted over a hydrophilic tipped guidewire (Sensor polytetrafluoroethylene (PTFE)-nitinol guidewire with hydrophilic tip; Boston Scientific, Marlborough, MA, USA) through the ureter with or without dilatation of the ureteric orifice if needed using balloon dilatation (Uromax Ultra; Boston Scientific). In most of our cases there was difficulty in passing the guidewire beyond the stone at initial attempts due to stone impaction. Laser fragmentation was done at the beginning to create a space for passing a safety guidewire, and then dusting of the rest of the stone was carried out. Stone fragmentation was performed using a holmium laser (Auriga XL 50-W holmium Laser, Boston Scientific) using 365- or 200-µm fibre, with energy applied at the settings of: 0.8–1 J/pulse, frequency 6–10 Hz, and long pulse duration. Flexible URS was used either from the start or if stone retropulsion occurred to reach the migrated fragments in the kidney. A JJ stent (5 F, 26 cm, Percuflex; Boston Scientific) was routinely placed at the end of the procedure.

### Group B, antegrade miniperc URS

Under general anaesthesia, all patients were placed in a prone position. Ultrasonography (US) was used to access the kidney with an 18-G Chiba needle, and then dye was injected to opacify the pelvi-calyceal system (PCS). Under fluoroscopic guidance an upper or middle calyceal puncture was made. A hydrophilic guidewire was introduced through the needle into the PCS, and then Teflon dilators were used to dilate the tract to 14 F. A ureteric access sheath (Navigator 13/11 F; Boston Scientific) was then inserted through the skin to the PCS over the guidewire. The flexible ureteroscope was introduced through the access sheath to reach the stone. Stone fragmentation was carried out using the same laser device with same settings as Group A. A JJ stent (5 F, 26 cm, Percuflex; Boston Scientific) was inserted in an antegrade fashion in all cases at the end of the procedure. At the end of the procedure the ureteric access sheath was removed without inserting a percutaneous tube.

All intraoperative and postoperative data were collected for statistical analysis. These data included operative time, which was defined in Group A as the time that elapsed from the start of introducing the instruments through the urethra until JJ-stent insertion; while in Group B operative time was defined as the time that elapsed from US-guided puncture until JJ-stent insertion. Fluoroscopy time and intraoperative complications were also reported.

Non-contrast CT was done after 2 weeks, before JJ-stent removal, for SFR assessment. Stone-free status was defined as the absence of fragments of >0.3 cm at the time of assessment. Postoperative complications and the need for auxiliary procedures were reported. The primary endpoint in this study was the SFR at 2 weeks, while the secondary endpoints included operative time, fluoroscopy time, lithotripsy time, postoperative complications, and hospital stay.

Data were analysed using the Statistical Package for the Social Sciences (SPSS®), version 20.0. (SPSS Inc., IBM Corp., Armonk, NY, USA). The Kolmogorov–Smirnov, Shapiro and D’Agstino tests were used to verify the normality of the distribution of variables. Comparisons between groups for categorical variables were assessed using the chi-square test (Fisher’s or Monte Carlo). The Student’s *t*-test was used to compare two groups for normally distributed quantitative variables and the Mann–Whitney test was used to compare between two groups for abnormally distributed quantitative variables. Significance of the obtained results was judged at the 5% level.

## Results

The study included 60 patients divided equally into the two groups, which were comparable in terms of demographic data and stone criteria, as shown in Table 1. Intraoperative parameters and postoperative outcomes are described in Table 2. Group B had a significantly higher SFR (83.3%) compared to Group A (60%). Residual fragments were managed by ESWL in both groups before JJ-stent removal. Both procedures were successfully carried out and patients were discharged the next day. All complications recorded were Grade I according to the Clavien–Dindo Classification. Four patients in Group B (13.3%) had bleeding of <150 mL, with no blood transfusion required. Six patients (20%) in Group A had guidewire trauma during laser firing with no total breakage. Postoperative complications were comparable with <50% having mild haematuria and mild fever. Postoperative pain was reported more in Group B, where 16 patients (53.3%) required intravenous analgesia and opioids compared to six patients (20%) in Group A.

**Table 1.** Patient’s demographics and stone criteria.

Variable	Group A RURS (*n* = 30)	Group B Antegrade miniperc URS (*n* = 30)	Test of significance	*P*
Age, years, mean (SD)	45.9 (12.5)	47.5 (11.7)	*t* = 0.490	0.626
Sex, *n* (%)				
Male	14 (46.7)	16 (53.3)	χ^2^ = 0.267	0.606
Female	16 (53.3)	14 (46.7)		
Stone criteria				
Size, cm, mean (SD)	1.35 (0.19)	1.33 (0.23)	*t* = 0.487	0.628
Density, HU, mean (SD)	979.4 (234.4)	871.4 (206.7)	*t* = 1.893	0.063
Laterality, *n* (%)				
Right	18 (60.0)	22 (73.3)	χ^2^ = 1.200	0.273
Left	12 (40.0)	8 (26.7)		
Site				
L2–L3	6 (20.0)	6 (20.0)	χ^2^ = 0.444	0.931
L3	8 (26.7)	10 (33.3)		
L3–4	10 (33.3)	8 (26.7)		
L4	6 (20.0)	6 (20.0)		

χ2: chi-square test; *t*: Student *t*-test; *P: P* value for comparison between the studied groups.

**Table 2.** Intraoperative variables and postoperative outcomes.

Variable	Group A RURS (*n* = 30)	Group B Antegrade miniperc URS (*n* = 30)	Test of significance	*P*
Operative time, min, mean (SD)	64.7 (17.7)	112.0 (15.3)	*t* = 11.098	<0.001*
Lithotripsy time, min, mean (SD)	39 (13.4)	38.3 (10.9)	*t* = 0.211	0.834
Fluoroscopy time, s, mean (range)	11 (8–16)	200 (160–300)	*U* = 0.00*	<0.001*
SFR, *n* (%)	18 (60.0)	25 (83.3)	χ^2^ = 4.022*	0.045*

χ2: chi-square test; *t*: Student *t*-test; *U*: Mann–Whitney *U*-test; *P: P* value for comparison between the studied groups.

*Statistically significant at *P* ≤ 0.05.

## Discussion

Treatment of large impacted proximal ureteric stones remains controversial. Both RURS and the antegrade approach represent effective treatment options, with both having their own pros and cons. RURS has the advantages of less invasiveness as it enter through a natural way, easier access to the stone, shorter operative time, less radiation exposure and shorter hospital stay; moreover, it is considered the first line of treatment according to American and European guidelines on ureteric stone management for stones measuring >1 cm [8]. On the other hand, it carries the disadvantage of decreased visualisation due to the narrow space around the stone with mucosal oedema leading to an increased incidence of complications, such as mucosal injury, ureteric wall perforation, instrument breakage, and stone retropulsion [9]. Stone retropulsion is a common problem in RURS during stone fragmentation, with a reported incidence of 28–60% for upper third ureteric stones. This leads to a decrease in the SFR and increases the need for secondary urological intervention [6].

The antegrade approach has proven to be a safe and effective treatment option with a higher SFR. It has the advantage of accessing the stone from the dilated wide upper ureter, with no risk of stone retropulsion and it provides better visualisation, and thus better stone fragmentation. On the other hand, some disadvantages have been noted regarding the renal puncture with increased radiation exposure, prolonged operative time, and postoperative hospital stay [10]. We applied the miniperc technique for our antegrade approach to minimise the limitations of this approach.

In our present study, 60 patients were included who were randomly divided equally into two groups, Group A (RURS) and Group B (miniperc antegrade URS). All the patients presented with a solitary large impacted upper third ureteric stone of >1 cm. In the present study, Group A had SFR of 60% significantly less than Group B (83.3%) after 2 weeks. Factors that improved the SFR in the antegrade group were the better stone visualisation, pressurised irrigation with no risk of retropulsion, and fragments washout during the procedure. Moufid et al. [11] conducted a retrospective study on 52 patients comparing between RURS using 9.5/8-F rigid URS and percutaneous antegrade URS using 20.8-F rigid nephroscope in the modified lateral position choosing impacted stones of ≥1.5 cm. The study reported a SFR for the retrograde approach of 66.7%, whereas for the antegrade approach it was 95.45% through a single tract in one session (*P* = 0.007). Their results were higher than the present study concerning the antegrade approach because they used ballistic lithotripsy and stone extraction, which was not applicable through the miniperc tract. Another reason may be because they used KUB for assessing stone clearance, which has low sensitivity unlike in our present study where we used non-contrast CT to assess stone clearance. Similar results confirming the superiority of antegrade approach were reported by Liu et al. [12], who had a 97.7% SFR in their antegrade group vs 82.2% in their retrograde group.

In the present study, the operative time was significantly shorter in Group A than Group B, at a mean (SD) of 64.7 (±17.7) vs 112.0 (±15.3) min. The reason for the prolonged operative time in Group B was the extra time taken for attaining the antegrade renal access including the puncture site, dilatation and reaching the stone. In the first few cases manipulation of the flexible URS to pass through the PUJ down to the ureter was rather difficult and time consuming, especially when we could not pass a wire down the ureter at the beginning of the renal puncture. In these cases, we spent time searching for the PUJ opening in the roomy pelvis, while the deflecting joint of the flexible URS was inside the ureteric access sheath. However, in all cases we succeeded to enter through the PUJ and reach the stone without instrumental trauma. In some cases the path from the ureteric access sheath down to the stone was not straight. In these cases, we were not able to reach the stone with flexible URS and then pass the laser fibre because this was going to risk injuring the inner channel of the flexible URS. In these cases, we had to preload the flexible URS with the laser fibre before passing it through the ureteric access sheath to the renal pelvis. While doing this, searching for the PUJ and manipulating the flexible URS with the laser fibre inside was more difficult and time consuming.

Different published studies have confirmed the longer operative time in the antegrade approach, e.g., Moufid et al. [11] who reported a higher mean operative time in the Perc URS group compared to the RURS group, at a mean (SD) of 66.5 (±21.7) vs. 52.13 (±17.3) min (*P* = 0.013). Similar results were found in the study conducted by Li et al. [13], who reported a mean (SD) operative time for percutaneous nephrolithotomy (PCNL) group at 108.76 (±19.36) vs 63.56 (±16.38) min for the RURS group (*P* < 0.05).

In the present study, no major complications occurred while minor complications were reported. Intraoperative bleeding of <150 mL occurred in four patients in Group B, while there was no bleeding in Group A. However, none of our patients required a blood transfusion. Guidewire injury occurred in six patients in Group A and none in Group B, reflecting the better visualisation during laser lithotripsy from the wide ureter above the stone were the pressure of the irrigant fluid can be increased without fear of stone retropulsion. Postoperative pain was reported in 16 patients (53.3%) in Group B mostly because of renal puncture.

Many studies have reported an overall low and similar rate of complications in both groups. Li et al. [13] reported comparable complication rates in both groups, with an increased incidence of ureteric perforation and stenosis in RURS group, while an increased incidence of haematuria and need for transfusion in the PCNL group. Sun et al. [14] reported bleeding in one patient (2.3%) for the antegrade procedure and ureteric injury in one patient (2.3%) for the retrograde procedure, with no statistically significance difference.

According to our experience during the present study, we believe that the miniperc antegrade approach is a promising alternative to the conventional retrograde approach for treating large impacted upper third ureteric stones. US-guided renal access in these patients was not difficult due to the presence of considerable hydronephrosis as a result of stone impaction. Dilatation of the tract to 14 F did not carry a risk of bleeding in most of the patients. The use of flexible URS via the antegrade route was rather difficult in the first few cases; however, it became easier with experience and the increasing learning curve. We believe that approaching the stone from above carries the advantages of better visualisation, no risk of stone retropulsion, less risk of mucosal or instrumental injury, and better fragments washout. The better visualisation is due to working from a wider space above the stone and the ability to push the irrigant fluid without risking migration of the stone. The pressurised irrigant helps better visualisation and also helps the washout of the fragments via the vacuum cleaner effect. The increased operative time and radiation exposure account for the main disadvantages of this approach. A limitation of the present study is that of being carried out in a single centre and also the limited number of patients. The primary endpoint in the present study identified a significantly higher SFR with the antegrade approach; we aim to increase the number of patients and enhance the learning curve to achieve a better operative time and avoid the identified limitations of the study.

## Conclusion

Antegrade miniperc URS for impacted upper ureteric stones is a feasible procedure that improves the SFR and lessens the need for secondary interventions, but at the expense of operative time and radiation exposure.
